# Fish and Cholera

**Published:** 1893-09-16

**Authors:** J. Lawrence-Hamilton

**Affiliations:** Late Honorary President Fishermen's 30, Sussex Square, Brighton. Federation


### Editor's Letter-Box.

[Our correspondents are reminded that proxility is a great bar to publication, and that brevity of style and conciseness of statement greatly facilitate early insertion.]

### FISH AND CHOLERA.

Sir,—Allow me to draw your attention to a fact which the present outbreaks of cholera at Grimsby and at Hull—our chief fishing ports—bring sadly into prominence. Upwards of thirty years' experience and observation in the chief fishing stations at home and abroad have convinced me that at too many of these food centres there is an increasing indifference to avoidable putrefactive filth. Altogether apart from the manner in which inoculation occurs, cholera, jail-fever, virulent small-pox, typhoid, typhus, leprosy, the plague, &c., have been always associated with avoidable filth. Dirty surroundings diminish our resistance to these infectious diseases — often preventable diseases — against which a healthier, stronger condition, obtainable by superior sanitation, becomes immune or free. Of course, avoidable filth tends to preserve the specific agents—the special bacteria and their products — which produce and propagate these hideous, but avoidable, diseases. The veteran scientist, Professor Dr. Virchow, when sent as a young man to investigate an epidemic outbreak, then suggested as a cure municipal reform with free action. Professor Dr. Koch, when recently reporting on the German cholera epidemic, considered it caused by contagion carried in a foul water supply, which we know has also introduced epidemic typhoid at Worthing. Like the ancient Egyptians among whom he dwelt, Moses considered certain kinds of "fish" the producer of "leprosy"—then skin diseases and skin rashes generally. Hence Jews are forbidden to eat oysters, shell-fish, and fish with fins or scales. In 1880 I pointed out the association of Astrakhan—the centre of the sturgeon and its caviare industries—with the plague which had recently ravaged Russia, &c. I again urge the necessity of making fresh fish imperishable, or always healthy, by the following methods of procedure: (1) On capture, immediate bleeding before blood clotting; (2) immediate gutting; (3) rapid cleaning, inside and outside, with abundant flowing cold (sea) water; (4) gradual cooling and dry air refrigeration.—I am, &c., J. Lawrence-Hamilton, M.R.C.S., Late Honorary President Fishermen's Federation.

